# The clinical application of metagenomic next-generation sequencing in infectious diseases at a tertiary hospital in China

**DOI:** 10.3389/fcimb.2022.957073

**Published:** 2022-12-19

**Authors:** Chuwen Wang, Danying Yan, Jiajia Huang, Naibin Yang, Jiejun Shi, Shou Pan, Gaoqiang Lin, Ying Liu, Yingying Zhang, Xueyan Bian, Qifa Song, Guoqing Qian

**Affiliations:** ^1^ School of Medicine, Ningbo University, Ningbo, Zhejiang, China; ^2^ Department of Infectious Diseases, Ningbo First Hospital, Ningbo University, Ningbo, Zhejiang, China; ^3^ Hangzhou DIAN Medical Laboratory, Hangzhou, China; ^4^ Vision Medicals Center for Infectious Diseases, Guangzhou, Guangdong, China; ^5^ Department of Nephrology, Ningbo First Hospital, Ningbo University, Ningbo, Zhejiang, China; ^6^ Medical Data Center, Ningbo First Hospital, Ningbo University, Ningbo, China

**Keywords:** infectious diseases, pathogens detection, metagenomic, next-generation sequencing, clinical diagnostic

## Abstract

**Background:**

Compared with traditional diagnostic methods (TDMs), rapid diagnostic methods for infectious diseases (IDs) are urgently needed. Metagenomic next-generation sequencing (mNGS) has emerged as a promising diagnostic technology for clinical infections.

**Methods:**

This retrospective observational study was performed at a tertiary hospital in China between May 2019 and August 2022. The chi-square test was used to compare the sensitivity and specificity of mNGS and TDMs. We also performed a subgroup analysis of the different pathogens and samples.

**Results:**

A total of 435 patients with clinical suspicion of infection were enrolled and 372 (85.5%) patients were finally categorized as the ID group. The overall sensitivity of mNGS was significantly higher than that of the TDMs (59.7% vs. 30.1%, *P* < 0.05). However, there was no significant difference in the overall specificity between the two methods (83.3% vs. 89.6%, *P* = 0.37). In patients with identified pathogens, the positive rates of mNGS for detecting bacteria (88.7%), fungi (87.9%), viruses (96.9%), and *Nontuberculous mycobacteria* (NTM; 100%) were significantly higher than those of TDMs (*P* < 0.05). The positive rate of mNGS for detecting *Mycobacterium tuberculosis* was not superior to that of TDMs (77.3% vs. 54.5%, *P* = 0.11). The sensitivity rates of mNGS for pathogen identification in bronchoalveolar lavage fluid, blood, cerebrospinal fluid, pleural fluid, and tissue were 72.6%, 39.3%, 37.5%, 35.0% and 80.0%, respectively.

**Conclusion:**

With the potential for screening multiple clinical samples, mNGS has an overall advantage over TDMs. It can effectively identify pathogens, especially those that are difficult to identify using TDMs, such as NTM, chlamydia, and parasites.

## 1 Introduction

Infectious diseases (IDs) have caused significant damage to social security and economic development (Nii-Trebi, 2017). With the emergence and development of antibiotics, the mortality rate of IDs has significantly decreased. However, the emergence of drug-resistant pathogens, especially multidrug resistant and pan-drug–resistant pathogens, such as *Klebsiella pneumoniae*, *Acinetobacter baumannii*, and *Pseudomonas aeruginosa* (Giamarellou and Poulakou, 2009; Munoz-Price et al., 2013), as well as new pathogens such as SARS-CoV-2 (Gorbalenya et al., 2020), has posed new challenges, emphasizing the importance of precision diagnosis (Simner et al., 2018).

Identification of pathogens in clinical infections has long relied on traditional culture, polymerase chain reaction (PCR) detection, and antigen/antibody immunological methods (Zheng et al., 2021). However, the etiology of suspected infections in hospitalized patients frequently remains unknown, resulting in prolonged hospitalization, misdiagnosis, and increased mortality or morbidity. Furthermore, the sensitivity of culture is limited by the empirical priority usage of antibiotics in clinical practice, and the results are time-consuming, particularly for the cultures of *Mycobacterium tuberculosis* (MTB) and fungi (Waterer and Wunderink, 2001; Mandell et al., 2007; Han et al., 2019). Traditional culture cannot be used for viral diagnosis (Gu et al., 2019). In addition, PCR detection and antigen/antibody immunological methods must be based on the genetic sequences or target proteins of known pathogens (Miao et al., 2018).

Metagenomic next-generation sequencing (mNGS) is a promising unbiased diagnostic tool that demonstrates the advantages of rapid, user-friendly data analysis tools, accurate databases, and culture-independent detection (Schlaberg et al., 2017; Simner et al., 2018). To date, several studies have revealed its effects and potential, which can be successfully applied to different sample types, such as cerebrospinal fluid (CSF), blood, bronchial alveolar lavage fluid (BALF), and tissues (Chen et al., 2020; Zhang et al., 2020; Guo et al., 2021). It is likely that, due to the limitations of mNGS, such as high cost, no uniform standards for experimental procedures, human background and background bacteria contamination (Gargis et al., 2016; Deurenberg et al., 2017; Han et al., 2019; Blauwkamp et al., 2019; Mitchell and Simner, 2019; Ramachandran and Wilson, 2020), its clinical applicability has lagged behind the research (Chiu and Miller, 2019).

In this study, to provide further evidence for the clinical application of mNGS, 435 patients with proven or suspected infections were analyzed to compare the sensitivity and specificity between mNGS and traditional diagnostic methods (TDMs) in identifying pathogens. Subgroup analyses of different pathogens and samples were also performed.

## 2 Methods

### 2.1 Study design

This retrospective study was performed at the Ningbo First Hospital, University of Ningbo, China. The requirement for written informed consent was waived, owing to the retrospective nature of this study. Patients with mNGS data were recruited, and the inclusion criteria were as follows: (1) age ≥ 18 years, (2) visit time from May 2019 to August 2022, and (3) proven or suspected infections. The exclusion criteria were as follows: (1) incomplete clinical data, including microbiological data, (2) failure to acquire a sufficient sample for mNGS analysis, and (3) a life expectancy of <48 h.

### 2.2 Metagenomic next-generation sequencing and analysis

#### 2.2.1 Specimens, nucleic acid extraction, and library generation

In this study, the sample type for mNGS was not limited, and clinical specimens were collected according to the standard procedures. The blood was placed in ethylenediaminetetraacetic acid tubes and transported at room temperature. The other samples were collected in sterile tubes and transported to a drikold. All samples were sent for mNGS within 24 h.

DNA extraction was conducted for each sample, and RNA extraction and reverse transcription were performed according to the clinical needs and the financial statuses of patients. DNA was extracted using the TIANamp Micro DNA Kit (Tiangen Biotech). For RNA extraction, total RNA was extracted using the QIAamp ViralRNA Kit and the Microbiome Kit (Qiagen). Ribosomal RNA was removed by the Ribo-Zero rRNA Removal Kit (Illumina). cDNA was generated using reverse transcriptase and deoxy-ribonucleoside triphosphates (dNTPs) (Thermo Fisher) (Amar et al., 2021). The total DNA or cDNA was subjected to library construction through the steps of fragment enzyme reaction, end-repairing, phosphorylation, A-tailing reaction, and adapter sequence connection by using the Nextera XT DNA Library Prep Kit (Illumina, San Diego, CA) (Miller et al., 2019). Library quality was assessed by a Qubit dsDNA HS Assay kit followed by a High Sensitivity DNA kit (Agilent) on an Agilent 2100 Bioanalyzer. Library pools were then loaded onto an Illumina Nextseq CN500 sequencer for 75 cycles of paired-end sequencing to generate approximately 20 million reads for each library. For negative controls, sterile deionized water was extracted alongside the specimens to serve as non-template controls (Li et al., 2018; Miller et al., 2019).

#### 2.2.2 Bioinformation pipeline

Trimmomatic was used to remove low-quality reads, adapter contamination, and duplicate reads, as well as those shorter than 50 bp (Xu et al., 2020). Low complexity reads were removed by K-complexity with default parameters (Bolger et al., 2014). Human sequence data were identified and excluded by mapping to a human reference genome (hg38) using Burrows-Wheeler Aligner software (Li and Durbin, 2009). The remaining sequence data were aligned to the current bacterial, virus, fungal, and protozoan databases (National Center for Biotechnology Information, ftp://ftp.ncbi.nlm.nih.gov/genomes). The database used in this study contained 9,694 bacterial species, 6,761 viral species, 1,551 fungal species, 305 parasites, 144 species of mycobacteria, and 107 mycoplasma/chlamydia related to human diseases.

#### 2.2.3 Interpretation of mNGS data

According to the published article, the criteria for mNGS-positive results were as follows (Miao et al., 2018):

1. Bacteria (except mycobacteria), viruses, and parasites: When the coverage rate of the microorganism was 10-fold higher than that of other microorganisms, the microorganism was identified as the pathogen.
2. Fungi: When the coverage rate of the fungus was five-fold higher than that of other fungi, it was identified as the pathogen.
3. MTB: MTB was considered positive when at least one read was mapped to either the species or genus levels.
4. Nontuberculous mycobacteria (NTM): NTM was considered positive when the mapping read number (genus or species level) was among the top 10 in the bacterial list.

### 2.3 Traditional diagnosis methods

TDMs include histopathological biopsy, microbial smear, microbial culture of various clinical samples (including body fluids, secretions and tissues), PCR detection, and antigen/antibody immunology.

### 2.4 Statistical analysis

The final diagnosis was independently determined by two physicians based on comprehensive clinical analysis, and a third physician was consulted to reach a consensus on the uncertainties. The chi-square test was used to compare the sensitivity and specificity between mNGS and TDMs. Statistical significance was set at *P* < 0.05. Data cleaning and analysis were performed using Microsoft Excel 2016 and R version 3.2.3. In addition, the figures were conducted in the R version 3.2.3 and Adobe Illustrator CC 2018.

## 3 Results

### 3.1 Patient and specimen characteristics

**Figure 1:**
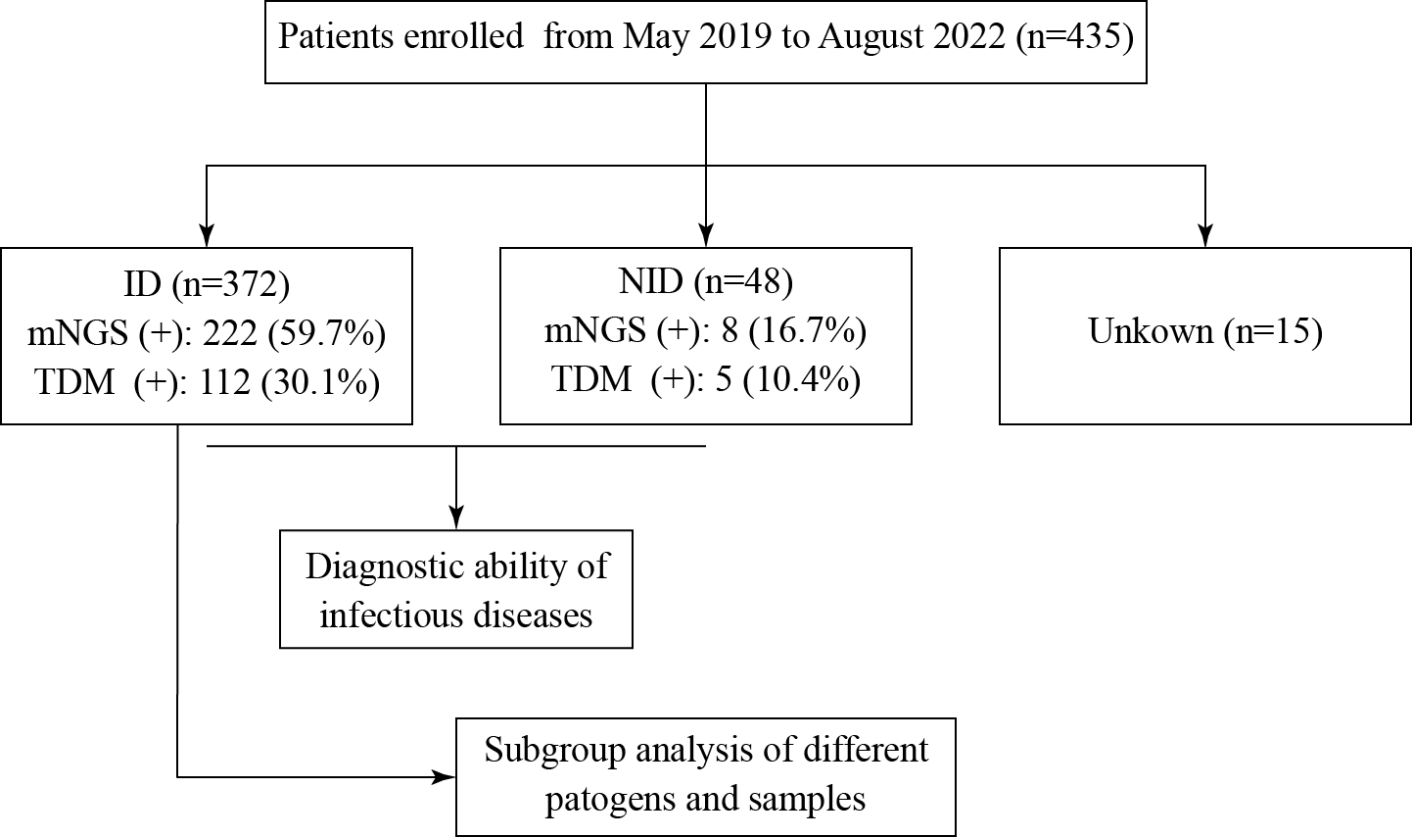
Flowchart of this study. A total of 435 patients were enrolled and divided into the ID, NID, and unknown groups. ID and NID patients were used to evaluate the diagnostic ability of mNGS and TDM to detect infection. ID, infectious disease; NID, non-infectious disease; mNGS, metagenomic next-generation sequencing; TDM, traditional diagnostic method.

**Figure 2:**
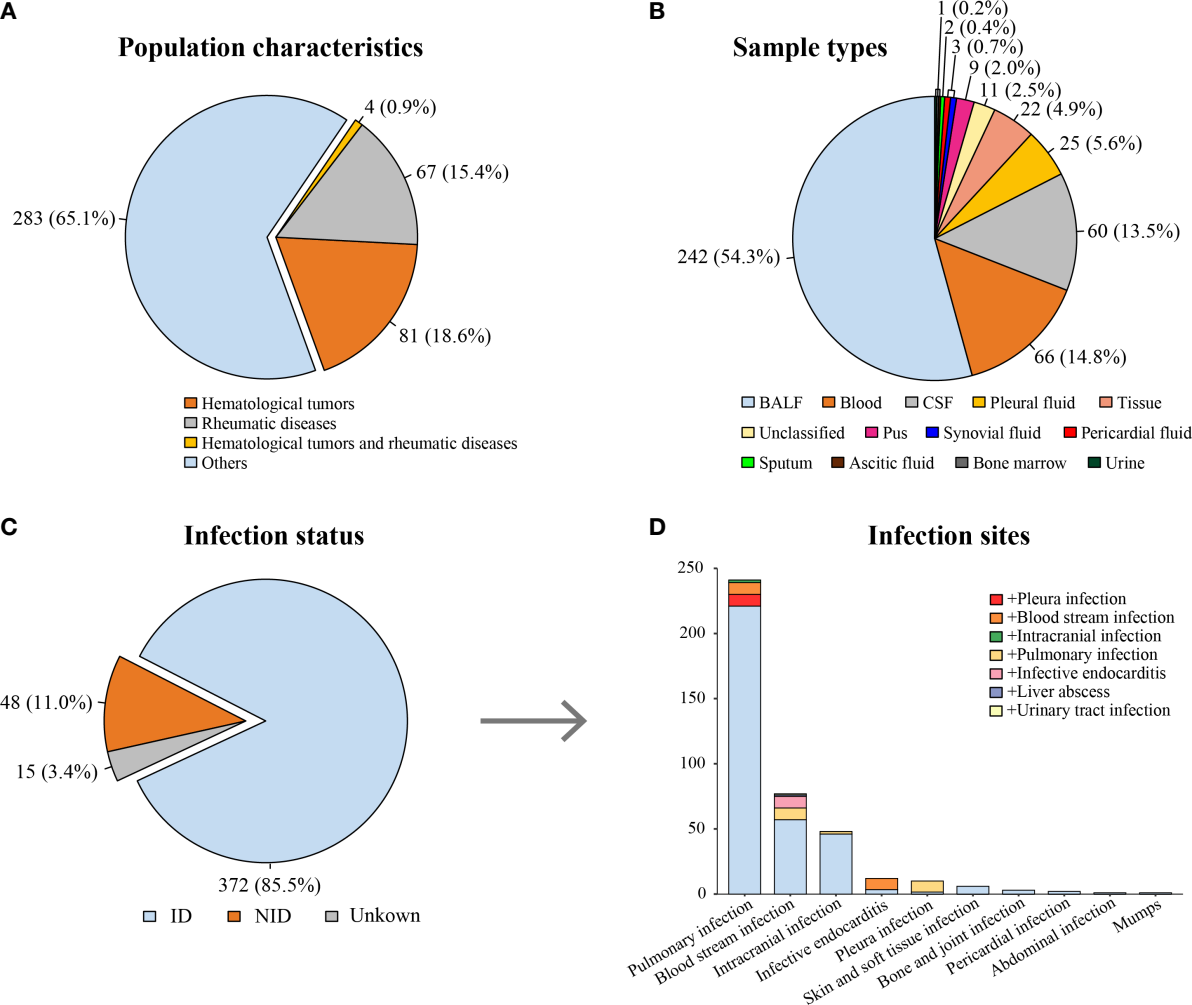
Characteristics of patients and specimens. **(A)** Proportion of patients suffering from hematological tumors or rheumatic diseases. **(B)** The distribution of specimens using for mNGS. **(C)** The distribution of infection status. **(D)** The distribution of infection sites in the ID group [the color block at the bottom of each column (light blue) indicates single site infection, whereas other color blocks indicate concurrent infection of other sites]. ID, infectious disease; NID, non-infectious disease; BALF, bronchial alveolar lavage fluid; CSF, cerebrospinal fluid.

According to the inclusion and exclusion criteria, 435 patients with clinical suspicion of infection were enrolled, and 446 specimens were screened using mNGS (**Figure 1**). The mean age of the patients was 57 years old (range, 19–91 years), and the male-to-female ratio was 1.25. Among them, 81 patients had hematological tumors (including myelodysplastic syndrome), 67 patients had rheumatic diseases, and four patients had both hematological tumors and rheumatic diseases (**Figure 2A**). After comprehensive evaluation, patients were finally divided into the ID group (372, 85.5%), the non-ID (NID) group (48, 11.0%), and the unknown group (15, 3.4%) (**Figure 2C**). Specimens were mainly BALF (242, 54.3%), blood (66, 14.8%), CSF (60, 13.5%), pleural fluid (25, 5.6%), and tissue (22, 4.9%) (**Figure 2B**).

### 3.2 Pathogen spectrum of the ID group

In the ID group, most patients (241, 64.8%) were diagnosed with pulmonary infection. In addition, 48 (12.9%) patients were diagnosed with intracranial infection, and 12 (3.2%) patients were diagnosed with infective endocarditis (IE) (**Figure 2D**). The most common infection was bacterial infection (124 of 372, 33.3%), caused by *Acinetobacter baumannii* (21 of 124, 16.9%), *Pseudomonas aeruginosa* (16 of 124, 12.9%), and *Klebsiella pneumoniae* (14 of 124, 11.3%). Fungal infection occurred in 66 patients, mainly by *Aspergillus* infection (38 of 66, 57.6%). There were 32 cases of viral infection (mainly Epstein–Barr virus), 31 cases of NTM infection, 22 cases of MTB infection, six cases of parasite infection, four cases of mycoplasma/chlamydia infection, and one case of rickettsia infection (**Supplementary Table 1**).

As shown in **Supplementary Table 2**, co-infection was detected in 37 patients, including six patients with hematological tumors and four patients with rheumatic diseases. Co-infection was mainly caused by fungi combined with other types of pathogens (26 patients), especially bacteria (14 patients).

### 3.3 The concordance between mNGS and TDMs

**Figure 3:**
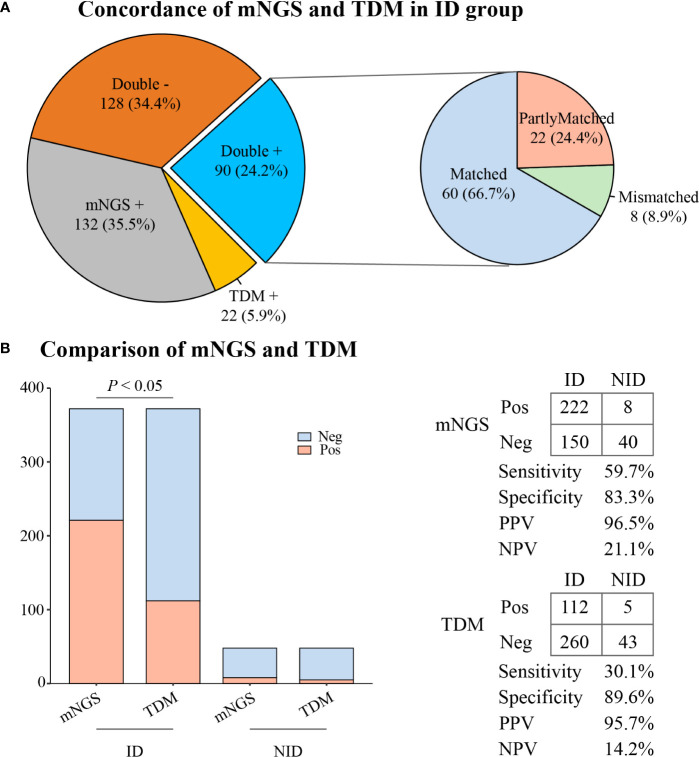
The concordance and diagnostic ability of mNGS and TDM. **(A)** Concordance of mNGS and TDM in the ID group. **(B)** Comparison of diagnostic ability between mNGS and TDM. mNGS, metagenomic next-generation sequencing; TDM, traditional diagnostic method; ID, infectious disease; NID, non-infectious disease; Pos, positive; Neg, negative; PPV, positive predictive value; NPV, negative predictive value.

In the ID group, the results of mNGS and TDMs were both positive in 90 (24.2%) cases and negative in 128 (34.4%) cases. Twenty-two (5.9%) patients only had positive results from the TDMs, and 132 (35.5%) patients only had positive results from mNGS. Of the 90 double-positive cases, 60 (66.7%) cases were completely matched, and eight (8.9%) cases were mismatched. Twenty-two (24.4%) cases were partially matched, which meant that there was at least one pathogen overlap between mNGS and TDMs (**Figure 3A**).

### 3.4 The diagnostic performance of mNGS and TDMs

As illustrated in **Figure 3B**, the overall sensitivity of mNGS was significantly higher than that of the TDMs (59.7% vs. 30.1%, *P* < 0.05). However, there was no significant difference in overall specificity between the two methods (83.3% vs. 89.6%, *P* = 0.37). The positive predictive value (PPV) and negative predictive value (NPV) of diagnosing ID by mNGS were 96.5% and 21.1%, respectively, with a positive likelihood ratio of 3.57 and a negative likelihood ratio of 0.48.

**Figure 4:**
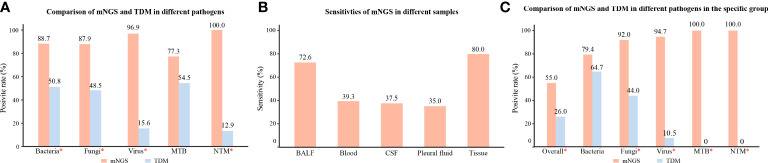
Subgroup analysis of different pathogens, samples, and specific group. **(A)** Comparison of positive rates of mNGS and TDM for different pathogens in patients with identified pathogens. **(B)** Sensitivity rates of mNGS in different specimens. **(C)** Comparison of positive rates of mNGS and TDM for different pathogens in pathogen-identified patients with hematological tumors or rheumatic diseases; *, *P* value was < 0.05. mNGS, metagenomic next-generation sequencing; TDM, traditional diagnostic method; ID, infectious disease; MTB, *Mycobacterium tuberculosis*; NTM, *Nontuberculous mycobacteria*; BALF, bronchial alveolar lavage fluid; CSF, cerebrospinal fluid.

In patients with identified pathogens (**Figure 4A**), the positive rates of mNGS for detecting bacteria (88.7%), fungi (87.9%), viruses (96.9%), and NTM (100%) were significantly higher than those of TDMs (*P* < 0.05). However, the positive rates of mNGS for detecting MTB were not superior to that of TDMs (77.3% vs. 54.5%, *P* = 0.11). The mNGS confirmed six cases of parasite infection, four cases of mycoplasma/chlamydia infection, two cases of *V. vulnificus* infection, and one case of rickettsia infection, which were not detected by the TDMs. On the other hand, mNGS missed 14 cases of bacterial infection, eight cases of fungal infection, five cases of MTB infection, and one case of viral infection, which were identified by the TDMs (**Supplementary Table 1**).

For the different samples (**Figure 4B**), the sensitivity of mNGS in BALF was 72.6%, which was significantly higher than that in blood (39.3%, *P* < 0.05). In addition, the sensitivity of mNGS in CSF, pleural fluid, and tissue was 37.5%, 35.0%, and 80.0%, respectively.

For the ID group with hematological tumors or rheumatic diseases (**Figure 4C**), the positive rates of mNGS for detecting pathogens (55.0%), fungi (92.0%), virus (94.7%), MTB (100%), and NTM (100%) were significantly higher than those of TDMs (*P* < 0.05). However, there was no significant difference in the specificity in detecting bacteria between the two methods (79.4% vs. 64.7%, *P* = 0.18).

### 3.5 Use of mNGS to diagnose IE

Twelve patients in the ID group were diagnosed with IE. Among them, the most common pathogen was *Streptococcus* (9 of 12, 75.0%), two cases were *Staphylococcus* infections and one case was a fungal infection. Eight patients had positive blood culture results, and one patient was diagnosed by pathological biopsy. All pathogens were detected by mNGS in valve tissue samples (**Table 1**).

**Table 1:** Analysis of patients with IE.

No.	Pathogen type	mNGS	TDM
1	Fungi	*Histoplasma capsulatum*	Fungus
2	Bacteria	*Streptococcus*	*Streptococcus*
3	Bacteria	*Streptococcus pharyngitis*	*Streptococcus pharyngitis*
4	Bacteria	*Staphylococcus aureus*	*Staphylococcus aureus*
5	Bacteria	*Streptococcus*	*Streptococcus*
137	Bacteria	*Staphylococcus warneri*	*Staphylococcus warneri*
200	Bacteria	*Streptococcus salivarius*	Neg
229	Bacteria	*Streptococcus mitis*	Neg
267	Bacteria	*Streptococcus sanguis*	*Streptococcus sanguis*
297	Bacteria	*Streptococcus*	*Streptococcus*
308	Bacteria	*Streptococcus sanguis*	*Streptococcus sanguis*
353	Bacteria	*Granulicatella adiacens*, *Streptococcus infantarius*	Neg

IE, infective endocarditis; mNGS, metagenomic next-generation sequencing; TDM, traditional diagnostic method; Neg, negative.

### 3.6 Use of mNGS to diagnose intracranial infection

Forty-eight patients were diagnosed with intracranial infection. The sensitivity of mNGS for detecting intracranial infections was 37.5%, which was significantly higher than that of TDMs (16.7%, *P* < 0.05). The mNGS detected eight cases of viral infection, six cases of bacterial infection, one case of MTB infection, one case of NTM with *Aspergillus* infection, one case of bacterial with *Aspergillus* infection, and one case of cryptococcus infection. However, mNGS missed two cases of bacterial infection and one case of MTB infection that were detected by the TDMs (**Supplementary Table 3**).

## 4 Discussion

This study reflects the actual performance of mNGS in a clinical setting. We compared the ability of mNGS to detect pathogens with that of TDMs, performed a subgroup analysis of different pathogens and samples, and concluded that mNGS can greatly improve the detection rate of pathogens and contribute to the diagnosis of infections with various rare or atypical pathogens, such as NTM, chlamydia, and parasite, in clinical practice.

Pathogenic identification is the key to the diagnosis and treatment of IDs. Routine diagnostics have been the first line of defense for doctors against IDs. However, traditional culture results are often affected by prior empirical infection treatment and sampling specifications, which greatly reduce the diagnostic sensitivity (Waterer and Wunderink, 2001; Mandell et al., 2007). Studies have shown that, even in patients with severe pulmonary infection, the detection rate of blood cultures is only 0% to 14% (Afshar et al., 2009; Zhang et al., 2019). In this study, we found that the overall sensitivity of TDMs, including histopathological biopsy, culture, PCR detection, and antigen/antibody immunology, was only 30.0%. Consistent with other studies (Chen et al., 2020; Huang et al., 2020), our study found that the overall sensitivity of mNGS was significantly higher than that of the TDMs (59.7% vs. 30.1%, *P* < 0.05). In addition, mNGS can identify pathogens that are difficult to identify using TDMs, such as NTM, chlamydia, and parasites. In this study, mNGS detected 31 cases of NTM infection, six cases of parasite infection, four cases of mycoplasma/chlamydia infection, and one case of rickettsia infection. In addition, mNGS also detected two cases of *V. vulnificus* infection, which enabled the timely diagnosis and treatment of patients and reduced the mortality.

However, owing to the high sensitivity of mNGS, false-positive results may occur. In this study, the false-positive rate for mNGS was 14.6%. Identifying human background and background bacterial contamination and correctly interpreting mNGS results remain a challenge. Each procedure, from specimen collection to sequencing, can lead to nucleic acid contamination (Gargis et al., 2016; Deurenberg et al., 2017; Han et al., 2019; Blauwkamp et al., 2019). Therefore, strict mNGS operation guidelines and template-free controls are needed for mNGS analysis, which can help reduce and filter out contaminated background readings when interpreting results (Breitwieser and Salzberg, 2020).

Although the sensitivity of mNGS is higher than that of TDMs, the results of mNGS may also be false negative (Miao et al., 2018; Duan et al., 2021). In this study, mNGS missed 14 cases of bacterial infection, eight cases of fungal infection, one case of viral infection, and five cases of MTB infection, which were identified by the TDMs. The sensitivity of mNGS for detecting MTB was not superior to that of TDMs (77.3% vs. 54.5%, *P* = 0.11). Some pathogens with hard cell walls, such as fungi, may reduce the efficiency of nucleic acid extraction, leading to the false negatives in mNGS (Han et al., 2019). Because of the intracellular growth characteristics of MTB, less nucleic acid is released outside the cell, which makes detection difficult (Doughty et al., 2014). A previous study showed that the sensitivity rates of mNGS, Xpert, culture, and smear to detect MTB were 59.9%, 69.0%, 59.9%, and 24.6%, respectively, and 79.6% overall (Liu et al., 2021). Therefore, to detect and treat tuberculosis as early as possible, a combination of mNGS and traditional methods is recommended.

mNGS has been used to detect pathogens in a variety of illnesses, with a focus on IE and intracranial infections. In this study, there were 12 patients developed IE. Blood culture identified eight patients, whereas mNGS identified all 12 patients. It has been reported that blood and valvular cultures are negative in 31% of IE cases (Habib et al., 2015). In some circumstances, the germs can be detected only before antibiotics administration (Koegelenberg et al., 2004). However, mNGS clearly overcomes the issue of detecting diseases that are not culturable (Cheng et al., 2018). According to a recent study of IE, the long-term subacute course of IE and prior use of antibiotics clearly lowered the positive rate of culture but did not significantly affect the positive rate of mNGS (Cai et al., 2021). More notably, valve mNGS facilitates the detection of the pathogen in the subacute course of IE. As a result, mNGS is critical in IE pathogen detection and antibiotic targeting therapy. CSF is a sterile fluid with a low host nucleic acid background (Kanjilal et al., 2019). Therefore, CSF is an excellent sample for the diagnosis of intracranial infection by mNGS. In our study, the sensitivity of mNGS in detecting intracranial infection was significantly higher than that of TDMs (37.5% vs. 16.7%, *P* < 0.05), indicating the value of mNGS in identifying pathogens of intracranial infection (Zhang et al., 2020; Xing et al., 2021).

mNGS is currently under improvement and exploration. Currently, DNA-based mNGS is being commonly used in clinical practice. Although DNA constitutes the genetic material of bacteria and fungi, RNA viruses also constitute a large proportion of infectious pathogens (Manso et al., 2017). RNA-based mNGS can reveal the entire “infectome” (RNA viruses, DNA viruses, bacteria, and eukaryotes), because all, except for prions, express RNA (Shi et al., 2018). Therefore, combining DNA and RNA sequencing has multiple advantages. However, compared with DNA, human RNA has higher abundance and complexity and is easily degraded, which results in higher requirements for sample transportation and storage (Zheng et al., 2021).

In this study, we not only evaluated the overall diagnosis ability of mNGS but also conducted a subgroup analysis of different pathogens and samples to provide further insights into the clinical application of mNGS. This study had several limitations. First, this was a single-center retrospective study. To minimize the potential bias in results based on the single-center research, we expanded the study sample as much as possible to obtain sufficient subgroup sample size. Second, we did not consider the effect of antibiotic use before admission on the results of mNGS and cultures, which might underestimate the sensitivity of culture and overestimate the difference of sensitivity between culture and mNGS. However, our results were basically consistent with other studies. Third, we did not consider specific interventions, such as steroids or biologic agents. However, we conducted the subgroup analysis of the specific populations, namely, patients with rheumatic diseases or patients with hematological tumors, who were potential users of steroids or biological agents. mNGS is a very promising technique, especially for difficult and rare infections. Multicenter studies with more clinical samples are needed to comprehensively test the efficacy of mNGS.

## 5 Conclusion

Although mNGS has the problem of background microbial contamination, it can be partially eliminated through negative quality control, and the results of mNGS can be interpreted comprehensively in combination with clinical practice. With the potential for multiple screening clinical samples, mNGS had the overall superior advantage over TDMs. It can efficiently identify pathogens, especially those that are difficult to identify using TDMs, such as NTM, chlamydia, and parasites.

## Data availability statement

The original contributions presented in the study are included in the article/**Supplementary Material**. Further inquiries can be directed to the corresponding authors.

## Ethics statement

The ethical approval was granted by Ningbo First Hospital ethical committee (2022RS055).

## Author contributions

CW and DY wrote the manuscript and performed data analysis. JH had taken responsibility of the data integrity. NY and JS had taken responsibility of the accuracy of data analysis. SP, GL, YL, and YZ were responsible for data collection. GQ, QS and XB edited the manuscript. All authors contributed to the article and approved the submitted version.
