# Supplementary material for: The clinical application of metagenomic next-generation sequencing in infectious diseases at a tertiary hospital in China
Source: Front Cell Infect Microbiol. 2022 Dec 19;12:957073. doi: 10.3389/fcimb.2022.957073 (PMC9806342; doi:10.3389/fcimb.2022.957073)
Supplement: Supplementary file 1 [file Table_1.pdf]

Supplementary Table 1 Details of enrolled patients

No	Sex	Age (y)	Specific group	Infection status	Infection sites	Pathogen type	Specimens for mNGS	mNGS	TDM	Matching degree
1	M	64	/	ID	Blood stream infection, IE	Fungi	Tissue	Histoplasma capsulatum	Fungus	Matched
2	M	25	/	ID	Blood stream infection, IE	Bacteria	Tissue	Streptococcus	Streptococcus	Matched
3	M	51	/	ID	Blood stream infection, IE	Bacteria	Tissue	Streptococcus pharyngitis	Streptococcus pharyngitis	Matched
4	F	68	/	ID	Blood stream infection, IE	Bacteria	Tissue	Staphylococcus aureus	Staphylococcus aureus	Matched
5	M	58	/	ID	Blood stream infection, IE	Bacteria	Tissue	Streptococcus	Streptococcus	Matched
6	M	43	/	ID	Pulmonary infection	Bacteria	BALF	Leuconostoc lactis	Neg	/
7	F	67	RD	ID	Pulmonary infection, Intracranial infection	Fungi, NTM	BALF, CSF	Aspergillus fumigatus, Mycobacterium abscessum	Neg	/
8	F	26	RD	ID	Pulmonary infection	/	BALF	Neg	Neg	/
9	F	67	RD	ID	Pulmonary infection	Fungi	BALF	Aspergillus	Neg	/
10	F	36	RD	ID	Pulmonary infection	Parasite	BALF	Pneumocystis jiroveci	Neg	/
11	F	69	/	ID	Pulmonary infection	Bacteria, Virus	BALF	Haemophilus influenzae, Streptococcus pneumoniae, Human herpesvirus 1	Neg	/
12	F	69	/	ID	Pulmonary infection	NTM	BALF	Mycobacterium intracellulare	Acid-fast bacilli	Matched
13	F	33	/	ID	Pulmonary infection	Parasite	BALF	Pneumocystis jiroveci	Neg	/

No	Sex	Age (y)	Specific group	Infection status	Infection sites	Pathogen type	Specimens for mNGS	mNGS	TDM	Matching degree
14	F	49	/	ID	Pulmonary infection	Bacteria, Mycoplasma, NTM	BALF	Haemophilus influenzae, Staphylococcus aureus, Mycoplasma hominis, Ureaplasma parvum, Mycobacterium abscessum	Neg	/
15	M	48	/	ID	Pulmonary infection	Virus	BALF	Human herpesvirus 5	Neg	/
16	M	72	HT	ID	Pulmonary infection	MTB	BALF	MTB	Neg	/
17	F	67	/	ID	Pulmonary infection	Fungi	BALF	Candida albicans	Candida albicans	Matched
18	M	67	/	ID	Pulmonary infection	Fungi	BALF	Aspergillus fumigatus	Aspergillus	Matched
19	F	32	RD	ID	Pulmonary infection	Bacteria	BALF	Pseudomonas aeruginosa	Pseudomonas aeruginosa	Matched
20	F	54	/	ID	Pulmonary infection	NTM	BALF	Mycobacterium intracellulare	Acid-fast bacilli	Matched
21	M	59	/	ID	Pulmonary infection	NTM	BALF	Mycobacterium intracellulare	Acid-fast bacilli	Matched
22	F	70	HT	ID	Pulmonary infection	Fungi	BALF	Cryptococcus neoformans	Cryptococcus neoformans	Matched
23	M	70	/	ID	Pulmonary infection	Bacteria	BALF	Klebsiella mutans	CRE	Matched
24	F	63	RD	ID	Pulmonary infection	Fungi	BALF	Aspergillus fumigatus	Aspergillus	Matched
25	M	60	/	ID	Pulmonary infection	Bacteria	BALF	Serratia marcescens	Serratia marcescens	Matched
26	F	73	RD	ID	Pulmonary infection	Fungi	BALF	Candida albicans	Fungus	Matched
27	M	61	/	ID	Pulmonary infection	NTM	BALF	Mycobacterium abscessum	Neg	/
28	M	69	/	NID	/	/	BALF	Neg	Neg	/
29	M	62	/	NID	/	/	BALF	Neg	Neg	/
30	F	56	RD	NID	/	/	BALF	Pos	Neg	/
31	M	67	/	NID	/	/	BALF	Pos	Neg	/
32	F	38	RD	ID	Pulmonary infection	NTM	BALF	Mycobacterium abscessum	Neg	/
33	M	85	/	NID	/	/	BALF	Neg	Pos	/

No	Sex	Age (y)	Specific group	Infection status	Infection sites	Pathogen type	Specimens for mNGS	mNGS	TDM	Matching degree
34	F	48	/	NID	/	/	BALF	Neg	Neg	/
35	M	58	RD	ID	Pulmonary infection	Bacteria	BALF	Neg	Klebsiella pneumoniae, Proteus mirabilis	/
36	F	59	/	ID	Pulmonary infection	Bacteria	BALF	Staphylococcus aureus	Neg	/
37	F	32	HT, RD	ID	Pulmonary infection	Virus	BALF, Pleural fluid	Human herpesvirus 7	Neg	/
38	M	59	/	NID	/	/	BALF	Neg	Neg	/
39	F	41	HT	ID	Pulmonary infection	Fungi	BALF	Candida tropicalis	Neg	/
40	F	48	RD	ID	Pulmonary infection	Bacteria	BALF	Haemophilus parainfluenzae	G-	Matched
41	F	48	RD	ID	Pulmonary infection	Bacteria	BALF	Enterobacter cloacae complex	Neg	/
42	M	75	RD	NID	/	/	BALF	Neg	Neg	/
43	F	37	RD	ID	Pulmonary infection	/	BALF	Neg	Neg	/
44	F	37	RD	ID	Pulmonary infection	/	BALF	Neg	Neg	/
45	F	64	/	NID	/	/	BALF	Neg	Pos	/
46	M	46	/	NID	/	/	BALF	Neg	Neg	/
47	F	56	/	ID	Pulmonary infection	/	BALF	Neg	Neg	/
48	M	55	RD	ID	Pulmonary infection	Bacteria	BALF	Haemophilus parainfluenzae	Neg	/
49	M	44	RD	NID	/	/	BALF	Pos	Neg	/
50	M	68	HT	ID	Pulmonary infection	/	BALF	Neg	Neg	/
51	F	63	HT	ID	Pulmonary infection	Bacteria, Virus	BALF	Pseudomonas aeruginosa, Human herpesvirus 5	Neg	/
52	M	68	HT	ID	Pulmonary infection	/	BALF	Neg	Neg	/
53	M	31	HT	ID	Pulmonary infection	/	BALF	Neg	Neg	/
54	F	68	RD	NID	/	/	BALF	Neg	Neg	/

No	Sex	Age (y)	Specific group	Infection status	Infection sites	Pathogen type	Specimens for mNGS	mNGS	TDM	Matching degree
55	M	50	/	ID	Pulmonary infection	Fungi, MTB	BALF	Aspergillus fumigatus	Aspergillus, Acid-fast bacilli	Partly matched
56	F	41	/	ID	Pulmonary infection	Bacteria	BALF	Haemophilus parainfluenzae	Neg	/
57	M	66	/	NID	/	/	BALF	Neg	Neg	/
58	M	76	/	ID	Pulmonary infection	MTB	Tissue	MTB	MTB	Matched
59	M	53	/	ID	Pulmonary infection	MTB	Tissue	MTB	MTB	Matched
60	F	32	/	ID	Pulmonary infection	Fungi, MTB	Tissue	MTB	Aspergillus, MTB	Partly matched
61	F	71	/	ID	Pulmonary infection	MTB	Tissue	Neg	MTB	/
62	M	74	RD	NID	/	/	Tissue	Neg	Pos	/
63	F	58	/	ID	Pulmonary infection	/	BALF, Tissue	Neg	Neg	/
64	M	65	/	NID	/	/	Tissue	Neg	Neg	/
65	M	66	RD	NID	/	/	Bone marrow	Neg	Neg	/
66	F	44	/	ID	Intracranial infection	Bacteria	CSF	Streptococcus constellatus	Neg	/
67	F	19	/	ID	Intracranial infection	Virus	CSF	Human herpesvirus 1	Neg	/
68	M	82	/	ID	Intracranial infection	Virus	CSF	Human herpesvirus 3	Neg	/
69	M	73	/	ID	Intracranial infection	Virus	CSF	Human herpesvirus 3	Human herpesvirus 3	Matched
70	M	20	/	ID	Intracranial infection	Virus	CSF	Human herpesvirus 3	Human herpesvirus 3	Matched
71	M	38	HT	ID	Intracranial infection	/	CSF	Neg	Neg	/
72	M	63	/	ID	Intracranial infection	/	CSF	Neg	Neg	/
73	F	76	/	ID	Intracranial infection	/	CSF	Neg	Neg	/
74	M	55	/	ID	Intracranial infection	Bacteria	CSF	Neg	Acinetobacter baumannii, Stenotrophomonas maltophilia	/

No	Sex	Age (y)	Specific group	Infection status	Infection sites	Pathogen type	Specimens for mNGS	mNGS	TDM	Matching degree
75	F	64	/	NID	/	/	CSF	Neg	Neg	/
76	M	71	/	ID	Intracranial infection	/	CSF	Neg	Neg	/
77	F	47	/	ID	Intracranial infection	/	CSF	Neg	Neg	/
78	F	26	/	ID	Intracranial infection	/	CSF	Neg	Neg	/
79	M	30	/	ID	Intracranial infection	/	CSF	Neg	Neg	/
80	F	58	/	ID	Intracranial infection	/	CSF	Neg	Neg	/
81	M	29	/	ID	Intracranial infection	/	CSF	Neg	Neg	/
82	M	50	/	ID	Intracranial infection	/	CSF	Neg	Neg	/
83	M	70	/	NID	/	/	CSF	Neg	Neg	/
84	M	57	/	NID	/	/	CSF	Neg	Neg	/
85	M	52	RD	ID	Skin and soft tissue infection	Virus	Pus	Human herpesvirus 4	Neg	/
86	M	47	/	ID	Pulmonary infection	/	Pleural fluid, Sputum	Neg	Neg	/
87	M	43	RD	ID	Pericardial infection	/	Pericardial fluid	Neg	Neg	/
88	M	51	/	ID	Pulmonary infection, Pleura infection	Bacteria	Pleural fluid	Porphyromonas endodontalis	Neg	/
89	M	68	/	ID	Pulmonary infection, Pleura infection	Bacteria	Pleural fluid	Staphylococcus aureus	Neg	/
90	F	66	/	NID	/	/	Pleural fluid	Neg	Pos	/
91	M	37	HT	NID	/	/	Pleural fluid	Neg	Neg	/
92	M	49	/	NID	/	/	Pleural fluid	Neg	Neg	/
93	M	43	/	NID	/	/	Pleural fluid	Neg	Neg	/
94	F	61	/	ID	Pleura infection	/	Pleural fluid	Neg	Neg	/
95	M	34	/	ID	Pulmonary infection	/	Pleural fluid	Neg	Neg	/

No	Sex	Age (y)	Specific group	Infection status	Infection sites	Pathogen type	Specimens for mNGS	mNGS	TDM	Matching degree
96	M	21	/	ID	Pulmonary infection, Pleura infection	MTB	Pleural fluid	Neg	MTB	/
97	M	84	/	ID	Pulmonary infection, Pleura infection	MTB	Pleural fluid	Neg	MTB	/
98	F	37	HT	ID	Pulmonary infection, Blood stream infection	Bacteria	BALF, Blood, Tissue	Enterococcus cecorum	Enterococcus cecorum	Matched
99	F	49	HT	ID	Blood stream infection	Virus	Blood	Human herpesvirus 5	Neg	/
100	M	63	/	ID	Blood stream infection	/	Blood	Neg	Neg	/
101	F	39	/	ID	Blood stream infection	Bacteria, Virus	Blood	Stenotrophomonas maltophilia, Human herpesvirus 1	Neg	/
102	M	25	/	ID	Blood stream infection	Parasite	Blood	leishmania infantum	leishmania infantum	Matched
103	F	58	HT	ID	Blood stream infection	Bacteria	Blood	Neg	Pseudomonas aeruginosa	/
104	F	66	HT	NID	/	/	Blood	Pos	Neg	/
105	F	88	/	ID	Blood stream infection	Bacteria	Blood	Neg	Klebsiella pneumoniae	/
106	F	72	/	ID	Pulmonary infection, Blood stream infection	Bacteria	BALF, Blood	Neg	Acinetobacter baumannii	/
107	M	55	/	ID	Blood stream infection	Bacteria	Blood	Neg	Acinetobacter baumannii	/
108	F	64	/	ID	Blood stream infection	/	Blood	Neg	Neg	/
109	M	59	/	ID	Blood stream infection	/	Blood	Neg	Neg	/
110	M	71	/	ID	Blood stream infection, Urinary tract infection	Bacteria	Blood, Urine	Enterobacter kobei	Neg	/
111	F	68	/	ID	Blood stream infection	/	Blood	Neg	Neg	/
112	M	50	/	ID	Blood stream infection	/	Blood	Neg	Neg	/

No	Sex	Age (y)	Specific group	Infection status	Infection sites	Pathogen type	Specimens for mNGS	mNGS	TDM	Matching degree
113	F	59	HT	ID	Blood stream infection	/	Blood	Neg	Neg	/
114	F	29	/	ID	Skin and soft tissue infection	Bacteria	Pus	Streptococcus pyogenes	Streptococcus pyogenes	Matched
115	M	24	RD	NID	/	/	CSF	Pos	Neg	/
116	M	68	/	ID	Intracranial infection	MTB	CSF	Neg	MTB	/
117	M	62	/	ID	Pulmonary infection	Bacteria, Fungi, NTM	BALF	Klebsiella pneumoniae, Mycobacteria intracellular, Mycobacterium chimerae	Aspergillus fumigatus	Partly matched
118	M	77	HT	ID	Pulmonary infection	/	Pleural fluid	Neg	Neg	/
119	M	65	/	ID	Pulmonary infection	/	BALF	Neg	Neg	/
120	M	53	HT	ID	Pulmonary infection	Virus	BALF	Leptosvirus	Neg	/
121	F	52	RD	Unkown	/	/	BALF	Neg	Pos	/
122	M	43	/	NID	/	/	BALF	Pos	Neg	/
123	M	74	/	ID	Pulmonary infection	Bacteria	Sputum	Legionella pneumophila	Neg	/
124	F	66	RD	ID	Pulmonary infection	Fungi	BALF	Candida	Candida	Matched
125	M	49	/	ID	Pulmonary infection	MTB	BALF	MTB	Neg	/
126	M	54	HT	NID	/	/	BALF	Neg	Neg	/
127	M	59	/	NID	/	/	BALF	Pos	Neg	/
128	M	56	/	ID	Pulmonary infection	Fungi	BALF	Neg	Aspergillus	/
129	F	48	/	ID	Pulmonary infection	/	BALF	Neg	Neg	/
130	M	23	HT	ID	Pulmonary infection	/	Pleural fluid	Neg	Neg	/
131	M	43	RD	ID	Pulmonary infection	Fungi	BALF	Candida albicans	Candida albicans	Matched
132	F	58	/	ID	Pulmonary infection	Fungi	BALF	Neg	Aspergillus	/
133	M	35	/	ID	Intracranial infection	Bacteria	CSF	Acinetobacter baumannii	Neg	/
134	M	53	/	ID	Blood stream infection	Bacteria	Blood	Neg	Acinetobacter baumannii	/

No	Sex	Age (y)	Specific group	Infection status	Infection sites	Pathogen type	Specimens for mNGS	mNGS	TDM	Matching degree
135	F	66	HT	ID	Pulmonary infection	/	BALF	Neg	Neg	/
136	F	54	/	ID	Pulmonary infection	Fungi	BALF	Aspergillus	Aspergillus	Matched
137	M	65	/	ID	Blood stream infection, IE	Bacteria	Tissue	Staphylococcus warneri	Staphylococcus warneri	Matched
138	M	29	RD	Unkown	/	/	Unclassified	Pos	Neg	/
139	M	62	HT	ID	Pulmonary infection	Virus	BALF	Human herpesvirus 5	Human herpesvirus 5	Matched
140	F	53	/	ID	Intracranial infection	Virus	CSF	Human herpesvirus 4	Human herpesvirus 4	Matched
141	F	83	/	ID	Pulmonary infection	Fungi	BALF	Aspergillus nidulans	Neg	/
142	M	71	RD	ID	Pulmonary infection	Bacteria	BALF	Acinetobacter baumannii, Stenotrophomonas maltophilia	Acinetobacter baumannii	Partly matched
143	F	58	/	ID	Pulmonary infection	Bacteria	BALF	Pseudomonas aeruginosa	Pseudomonas aeruginosa	Matched
144	F	50	RD	ID	Pulmonary infection	/	BALF	Neg	Neg	/
145	M	63	/	ID	Pulmonary infection	MTB	BALF	MTB	MTB	Matched
146	M	79	HT	ID	Pulmonary infection	Bacteria	BALF	Neg	G, coccus	/
147	F	59	/	ID	Intracranial infection	Bacteria	CSF	Streptococcus gordonii	Neg	/
148	F	78	/	ID	Pulmonary infection	Fungi, MTB	BALF	Cryptococcus neoformans, MTB	Acid-fast bacilli	Partly matched
149	F	42	/	ID	Pulmonary infection	/	BALF	Neg	Neg	/
150	F	74	RD	ID	Pulmonary infection	Bacteria	BALF	Neg	G- bacilli	/
151	M	44	/	ID	Pulmonary infection	/	BALF	Neg	Neg	/
152	F	38	/	NID	/	/	BALF	Neg	Neg	/
153	F	64	/	ID	Pulmonary infection	NTM	Unclassified	Mycobacterium abscessum	Neg	/
154	M	53	/	ID	Pulmonary infection	/	BALF	Neg	Neg	/

No	Sex	Age (y)	Specific group	Infection status	Infection sites	Pathogen type	Specimens for mNGS	mNGS	TDM	Matching degree
155	F	69	HT	ID	Pulmonary infection	Bacteria	BALF	Enterococcus faecalis	Enterococcus faecalis	Matched
156	M	46	/	ID	Intracranial infection	Virus	CSF	Human herpesvirus 4	Neg	/
157	M	56	HT	ID	Pulmonary infection	Fungi	BALF	Aspergillus fumigatus	Neg	/
158	M	65	/	ID	Pulmonary infection	Bacteria	BALF	Porphyromonas gingivalis, actinomycetes	Neg	/
159	M	79	HT	ID	Blood stream infection	/	Blood	Neg	Neg	/
160	M	37	/	ID	Pulmonary infection	/	Unclassified	Neg	Neg	/
161	F	63	HT	ID	Pulmonary infection	Virus	BALF	Human herpesvirus 1	Neg	/
162	F	66	/	ID	Bone and joint Infection	/	Synovial fluid	Neg	Neg	/
163	F	73	RD	ID	Pulmonary infection	Fungi	BALF	Candida albicans	Candida albicans	Matched
164	M	66	/	ID	Pulmonary infection	MTB	BALF	MTB	MTB	Matched
165	F	73	/	ID	Pulmonary infection	Bacteria	BALF	Stenotrophomonas maltophilia, Acinetobacter baumannii	Acinetobacter baumannii	Partly matched
166	F	67	RD	ID	Pulmonary infection	Virus	BALF	Human herpesvirus 4	Neg	/
167	F	52	HT	ID	Pulmonary infection	Bacteria	BALF	Pseudomonas aeruginosa	Pseudomonas aeruginosa	Matched
168	M	60	HT	ID	Blood stream infection	/	Blood	Neg	Neg	/
169	M	31	HT	Unkown	/	/	BALF	Neg	Neg	/
170	M	47	/	ID	Bone and joint Infection	Bacteria	Synovial fluid	Staphylococcus aureus	Staphylococcus aureus	Matched
171	M	33	/	NID	/	/	Blood	Neg	Neg	/
172	M	49	RD	ID	Pulmonary infection	/	Pleural fluid	Neg	Neg	/
173	M	34	/	ID	Pulmonary infection	Fungi	BALF	Scedosporium apiospermum	Neg	/
174	M	67	HT	Unkown	/	/	BALF	Pos	Pos	/
175	M	53	/	ID	Intracranial infection	Bacteria	CSF	Lactococcus canadensis, Streptococcus gordon	Acinetobacter baumannii	Mismatched

No	Sex	Age (y)	Specific group	Infection status	Infection sites	Pathogen type	Specimens for mNGS	mNGS	TDM	Matching degree
176	F	22	/	ID	Pulmonary infection	/	Pleural fluid	Neg	Neg	/
177	F	52	/	ID	Intracranial infection	Bacteria	CSF	Neg	Citrobacter braakii	/
178	M	67	RD	ID	Pulmonary infection	/	BALF	Neg	Neg	/
179	F	51	HT	ID	Blood stream infection	Rickettsia	Blood	Rickettsia felis, Rickettsia australis	Neg	/
180	F	49	/	ID	Pulmonary infection	/	BALF	Neg	Neg	/
181	F	71	/	ID	Pulmonary infection	/	BALF	Neg	Neg	/
182	F	43	HT	Unkown	/	/	Blood	Neg	Neg	/
183	F	51	RD	ID	Blood stream infection	/	Blood	Neg	Neg	/
184	F	53	/	ID	Intracranial infection	/	CSF	Neg	Neg	/
185	M	59	/	ID	Pulmonary infection	/	BALF	Neg	Neg	/
186	F	64	HT	ID	Pulmonary infection	Fungi	BALF	Aspergillus	Neg	/
187	M	65	/	ID	Pulmonary infection	Fungi, parasite	BALF	Pneumocystis yersinii	Cryptococcus lorentus	Mismatched
188	F	79	/	ID	Pulmonary infection	NTM	BALF	Mycobacterium intracellular, Mycobacterium chimerae, Mycobacterium avium	Neg	/
189	F	54	RD	ID	Bone and joint Infection	MTB	Pus	MTB	Neg	/
190	F	54	/	ID	Pulmonary infection	Fungi, NTM	BALF	Aspergillus, Mycobacterium avium	Aspergillus	Partly matched
191	M	25	/	ID	Intracranial infection	Fungi	CSF	Cryptococcus neoformans	Cryptococcus neoformans	Matched
192	F	52	HT	ID	Blood stream infection	/	Blood	Neg	Neg	/
193	M	45	/	ID	Pulmonary infection	/	BALF	Neg	Neg	/
194	F	79	RD	ID	Pulmonary infection	NTM	BALF	Mycobacterium abscessum	Neg	/

No	Sex	Age (y)	Specific group	Infection status	Infection sites	Pathogen type	Specimens for mNGS	mNGS	TDM	Matching degree
195	F	57	RD	ID	Pulmonary infection	/	BALF	Neg	Neg	/
196	M	50	HT	ID	Blood stream infection	Bacteria	Blood	Enterobacter cloacae	Enterobacter hormaechei	Mismatched
197	M	67	/	ID	Pulmonary infection	Chlamydia	BALF	Chlamydia psittaci	Neg	/
198	M	30	/	ID	Pulmonary infection	/	BALF	Neg	Neg	/
199	M	68	/	Unkown	/	/	Blood	Pos	Neg	/
200	M	52	/	ID	IE	Bacteria	Tissue	Streptococcus salivarius	Neg	/
201	M	59	/	ID	Intracranial infection	/	CSF	Neg	Neg	/
202	F	82	/	ID	Intracranial infection	MTB	CSF	MTB	Neg	/
203	M	57	/	ID	Pulmonary infection	Bacteria, Fungi	BALF	Stenotrophomonas maltophilia, Candida albicans	Neg	/
204	M	64	/	ID	Pulmonary infection	NTM	BALF	Mycobacterium abscessum	Neg	/
205	M	51	/	ID	Intracranial infection	/	CSF	Neg	Neg	/
206	M	56	HT	ID	Blood stream infection	/	Blood	Neg	Neg	/
207	F	37	/	ID	Blood stream infection	/	Blood	Neg	Neg	/
208	M	86	/	ID	Pulmonary infection	MTB	BALF	MTB	Neg	/
209	F	62	/	NID	/	/	Unclassified	Neg	Neg	/
210	F	25	HT	ID	Blood stream infection	Virus, Fungi	Blood	Human herpesvirus 4	Aspergillus	Mismatched
211	M	70	/	ID	Pulmonary infection, Intracranial infection	/	BALF, CSF	Neg	Neg	/
212	F	79	/	ID	Blood stream infection	/	Blood	Neg	Neg	/
213	F	29	/	ID	Intracranial infection	/	CSF	Neg	Neg	/
214	F	54	HT	ID	Pulmonary infection	NTM	Pleural fluid	Mycobacterium Tuscany	Neg	/
215	M	44	/	ID	Intracranial infection	/	CSF	Neg	Neg	/

No	Sex	Age (y)	Specific group	Infection status	Infection sites	Pathogen type	Specimens for mNGS	mNGS	TDM	Matching degree
216	M	22	/	ID	Intracranial infection	/	CSF	Neg	Neg	/
217	M	64	/	ID	Pulmonary infection	Bacteria, Fungi	BALF	Haemophilus influenzae, Klebsiella pneumoniae, Aspergillus	Klebsiella pneumoniae	Partly matched
218	M	58	RD	ID	Pulmonary infection	/	BALF	Neg	Neg	/
219	M	65	/	Unkown	/	/	BALF	Neg	Neg	/
220	M	80	HT	ID	Pulmonary infection	/	BALF	Neg	Neg	/
221	F	64	RD	ID	Pulmonary infection	/	BALF	Neg	Neg	/
222	M	62	HT	ID	Pulmonary infection	Bacteria	BALF	Neg	Klebsiella pneumoniae	/
223	M	37	/	ID	Intracranial infection	/	CSF	Neg	Neg	/
224	M	57	/	ID	Pulmonary infection	Bacteria	BALF	Staphylococcus aureus	Neg	/
225	M	59	/	ID	Pulmonary infection	MTB	BALF	MTB	Neg	/
226	M	82	/	ID	Pulmonary infection	Bacteria	BALF	Pseudomonas aeruginosa	Pseudomonas aeruginosa	Matched
227	F	60	RD	ID	Pulmonary infection	Parasite	BALF	Pneumocystis jiroveci	Neg	/
228	M	62	/	ID	Pulmonary infection	/	BALF	Neg	Neg	/
229	F	60	/	ID	IE	Bacteria	Tissue	Streptococcus mitis	Neg	/
230	M	45	/	ID	Pulmonary infection	Bacteria	BALF	Bordetella pertussis	Neg	/
231	F	65	/	ID	Pulmonary infection	MTB	BALF	MTB	Neg	/
232	F	24	/	ID	Pulmonary infection, Pleura infection	Bacteria	Pleural fluid	Porphyromonas endodontalis	Neg	/
233	F	34	/	ID	Intracranial infection	Bacteria	CSF	Acinetobacter baumannii	Neg	/
234	F	56	/	ID	Pulmonary infection	/	BALF	Neg	Neg	/
235	M	35	/	ID	Intracranial infection	Virus	CSF	Human herpesvirus 3	Neg	/
236	M	51	/	Unkown	/	/	CSF	Neg	Pos	/

No	Sex	Age (y)	Specific group	Infection status	Infection sites	Pathogen type	Specimens for mNGS	mNGS	TDM	Matching degree
237	F	59	/	ID	Pulmonary infection	Fungi	BALF	Cryptococcus neoformans	Neg	/
238	M	28	/	ID	Pulmonary infection	Chlamydia	BALF	Chlamydia psittaci	Neg	/
239	M	47	HT	ID	Blood stream infection	/	Blood	Neg	Neg	/
240	M	91	/	ID	Pulmonary infection	Bacteria, NTM	BALF	Acinetobacter baumannii, Pseudomonas aeruginosa, Mycobacterium abscessum	Acinetobacter baumannii	Partly matched
241	M	63	HT	ID	Pulmonary infection	/	Unclassified	Neg	Neg	/
242	M	65	/	ID	Intracranial infection	/	CSF	Neg	Neg	/
243	F	75	/	ID	Pulmonary infection	Bacteria	BALF	Acinetobacter baumannii	Acinetobacter baumannii	Matched
244	M	68	/	ID	Pulmonary infection	/	BALF	Neg	Neg	/
245	M	72	/	NID	/	/	Tissue	Neg	Neg	/
246	M	64	/	ID	Pulmonary infection	/	BALF	Neg	Neg	/
247	M	66	/	ID	Pulmonary infection	Bacteria	BALF	Neg	Escherichia coli	/
248	F	69	/	ID	Pulmonary infection	MTB	BALF	MTB	Neg	/
249	M	47	HT	ID	Pulmonary infection	/	Pleural fluid	Neg	Neg	/
250	F	68	/	ID	Pulmonary infection	NTM	BALF	Intracellular mycobacterium	Neg	/
251	F	66	HT	ID	Pericardial infection	/	Pericardial fluid	Neg	Neg	/
252	F	62	/	ID	Pulmonary infection, Blood stream infection	Bacteria	BALF	Klebsiella pneumoniae	Klebsiella pneumoniae	Matched
253	M	74	/	ID	Pulmonary infection	Virus, Fungi	BALF	Human herpesvirus 4, Aspergillus flavus	Neg	/
254	F	66	/	ID	Intracranial infection	/	CSF	Neg	Neg	/
255	M	55	/	NID	/	/	BALF	Neg	Neg	/

No	Sex	Age (y)	Specific group	Infection status	Infection sites	Pathogen type	Specimens for mNGS	mNGS	TDM	Matching degree
256	F	62	/	ID	Pulmonary infection	Bacteria	BALF	Acinetobacter baumannii, Burkholderia neoonion	Neg	/
257	M	65	/	ID	Pulmonary infection	Bacteria	BALF	Haemophilus influenzae, Constellation streptococcus, Intermediate streptococcus	Neg	/
258	M	41	/	ID	Intracranial infection	/	CSF	Neg	Neg	/
259	F	58	/	NID	/	/	BALF	Neg	Neg	/
260	M	68	/	ID	Pulmonary infection	/	BALF	Neg	Neg	/
261	F	65	HT	ID	Blood stream infection	/	Blood	Neg	Neg	/
262	F	63	HT	ID	Blood stream infection	Virus	Blood	Human herpesvirus 5	Neg	/
263	M	33	/	ID	Pulmonary infection	Bacteria, Virus, NTM, Parasite	BALF	Serratia marcescens, Mycobacterium abscessum, Pneumocystis yersinii, Human herpesvirus type 4	Neg	/
264	F	28	/	ID	Pulmonary infection	/	BALF	Neg	Neg	/
265	M	66	HT	ID	Blood stream infection	Bacteria	Blood	Legionella pneumophila	Neg	/
266	F	51	HT	Unkown	/	/	BALF	Neg	Neg	/
267	F	24	/	ID	Blood stream infection, IE	Bacteria	Tissue	Streptococcus sanguis	Streptococcus sanguis	Matched
268	M	51	RD	NID	/	/	BALF	Neg	Neg	/
269	M	60	/	NID	/	/	CSF	Neg	Neg	/
270	M	41	/	NID	/	/	CSF	Neg	Neg	/
271	M	23	HT	ID	Blood stream infection	/	Blood	Neg	Neg	/
272	M	42	/	ID	Blood stream infection	Bacteria	Blood	Vibrio vulnificus	Neg	/

No	Sex	Age (y)	Specific group	Infection status	Infection sites	Pathogen type	Specimens for mNGS	mNGS	TDM	Matching degree
273	M	60	HT	ID	Pulmonary infection	Bacteria	BALF	Enterobacter hormaechei, Enterobacter cloacae, Burkholderia polyphagia	Enterobacter cloacae	Partly matched
274	F	55	/	ID	Blood stream infection, Liver abscess	Bacteria	Pus	Klebsiella pneumoniae	Klebsiella pneumoniae	Matched
275	F	59	/	ID	Pulmonary infection	Bacteria	BALF	Pseudomonas aeruginosa	Neg	/
276	F	47	HT	NID	/	/	CSF	Neg	Neg	/
277	F	63	/	ID	Pulmonary infection	Bacteria, NTM	BALF	Stenotrophomonas maltophilia, Mycobacterium avium	Neg	/
278	F	52	RD	ID	Pulmonary infection	Fungi	BALF	Aspergillus flavus	Neg	/
279	M	67	/	ID	Pulmonary infection	/	BALF	Neg	Neg	/
280	M	55	/	ID	Intracranial infection	/	CSF	Neg	Neg	/
281	F	37	/	ID	Pulmonary infection	MTB	BALF	MTB	Neg	/
282	M	53	/	ID	Pulmonary infection	Virus	BALF	Human herpes virus type 4	Neg	/
283	M	70	/	ID	Pulmonary infection	Fungi	BALF	Candida albicans	Candida albicans	Matched
284	F	53	/	ID	Pulmonary infection	Bacteria	BALF	Haemophilus influenzae	Neg	/
285	M	62	HT	ID	Pulmonary infection	Bacteria	BALF	Serratia marcescens	Neg	/
286	M	66	/	ID	Pulmonary infection	/	BALF	Neg	Neg	/
287	M	43	/	ID	Blood stream infection	/	Blood	Neg	Neg	/
288	M	79	/	ID	Pulmonary infection	Bacteria	BALF	Escherichia coli, Klebsiella heterophila	Escherichia coli	Partly matched
289	M	59	HT	ID	Blood stream infection	Virus	Blood	Human herpesvirus 4	Neg	/
290	F	67	HT	ID	Blood stream infection	Virus	Blood	Human herpesvirus 5	Neg	/

No	Sex	Age (y)	Specific group	Infection status	Infection sites	Pathogen type	Specimens for mNGS	mNGS	TDM	Matching degree
291	F	66	HT	ID	Pulmonary infection, Blood stream infection	Fungi	BALF, Blood	Black Rhizopus brevis	Neg	/
292	F	49	RD	ID	Pulmonary infection	/	Pleural fluid	Neg	Neg	/
293	M	79	RD	ID	Pulmonary infection	Bacteria	BALF	Enterobacter aeruginosa, Klebsiella pneumoniae	Neg	/
294	F	57	HT	ID	Blood stream infection	/	Blood	Neg	Neg	/
295	M	25	/	ID	Pulmonary infection	/	BALF	Neg	Neg	/
296	M	88	/	ID	Blood stream infection	/	Blood	Neg	Neg	/
297	F	59	/	ID	Blood stream infection, IE	Bacteria	Tissue	Streptococcus	Streptococcus	Matched
298	M	78	/	ID	Pulmonary infection	Bacteria	BALF	Klebsiella aerogenes	Neg	/
299	F	64	/	ID	Blood stream infection	/	Blood	Neg	Neg	/
300	F	75	/	ID	Pulmonary infection, Blood stream infection	Bacteria, NTM	BALF, Blood	Burkholderia neoonion, Elizabethania, Mycobacterium abscessum	Elizabethania	Partly matched
301	F	65	/	ID	Pulmonary infection	Bacteria	BALF	Pseudomonas aeruginosa	Neg	/
302	F	55	/	ID	Pulmonary infection	Bacteria	BALF	Klebsiella pneumoniae, Elizabeth meningitidis septicemia	Klebsiella pneumoniae	Partly matched
303	F	55	/	ID	Pulmonary infection	Fungi	BALF	Cryptococcus neoformans	Neg	/
304	F	78	/	ID	Pulmonary infection	Bacteria	BALF	Aeromonas veronii	Neg	/
305	M	31	RD	Unkown	/	/	Synovial fluid	Neg	Neg	/
306	F	65	RD	ID	Pulmonary infection	NTM	BALF	Mycobacterium abscessum	Neg	/
307	F	58	/	ID	Pulmonary infection	Bacteria	BALF	Pseudomonas aeruginosa	Neg	/

No	Sex	Age (y)	Specific group	Infection status	Infection sites	Pathogen type	Specimens for mNGS	mNGS	TDM	Matching degree
308	M	49	/	ID	Blood stream infection, IE	Bacteria	Tissue	Streptococcus sanguis	Streptococcus sanguis	Matched
309	F	54	HT	ID	Blood stream infection	Fungi	Blood	Aspergillus flavus, Aspergillus oryzae	Neg	/
310	M	73	/	Unkown	/	/	BALF	Pos	Pos	/
311	M	84	RD	ID	Skin and soft tissue infection	Fungi	Pus	Exophiala spinifera	Neg	/
312	F	69	/	ID	Pulmonary infection	Bacteria	BALF	Pseudomonas aeruginosa	Pseudomonas aeruginosa	Matched
313	F	89	/	ID	Pulmonary infection	Bacteria, Fungi	BALF	Stenotrophomonas maltophilia, Pseudomonas aeruginosa, Aspergillus fumigatus	Pseudomonas aeruginosa	Partly matched
314	F	72	RD	ID	Pulmonary infection	/	Unclassified	Neg	Neg	/
315	M	72	/	ID	Pulmonary infection	/	Unclassified	Neg	Neg	/
316	M	37	/	ID	Pulmonary infection	/	Unclassified	Neg	Neg	/
317	F	43	RD	ID	Pulmonary infection	Fungi	BALF	Cryptococcus neoformans	Cryptococcus neoformans	Matched
318	M	73	/	ID	Pulmonary infection	Bacteria, Fungi	BALF	Acinetobacter baumannii, Aspergillus fumigatus, Aspergillus oryzae, Aspergillus flavus	Acinetobacter baumannii	Partly matched
319	F	66	/	ID	Pulmonary infection	Fungi, NTM	BALF	Mycobacterium kansasii, Aspergillus terreus, Aspergillus fumigatus	Acid-fast bacilli, Fungus	Matched
320	M	59	HT	ID	Pulmonary infection	/	Unclassified	Neg	Neg	/
321	F	37	/	ID	Pulmonary infection, Blood stream infection	Bacteria	BALF	Staphylococcus aureus	Staphylococcus aureus	Matched

No	Sex	Age (y)	Specific group	Infection status	Infection sites	Pathogen type	Specimens for mNGS	mNGS	TDM	Matching degree
322	M	51	/	ID	Intracranial infection	/	CSF	Neg	Neg	/
323	F	39	RD	ID	Intracranial infection	/	CSF	Neg	Neg	/
324	M	36	/	ID	Intracranial infection	/	CSF	Neg	Neg	/
325	F	57	/	NID	/	/	BALF	Neg	Neg	/
326	F	79	HT	ID	Blood stream infection	Fungi	Blood	Candida albicans	Candida albicans	Matched
327	M	63	RD	ID	Pulmonary infection	Bacteria	BALF	Legionella pneumophila	Neg	/
328	M	67	RD	ID	Pulmonary infection, Pleura infection	Bacteria	Pleural fluid	Sphingomonas spinosa	Neg	/
329	M	79	/	ID	Pulmonary infection, Blood stream infection	Bacteria	BALF	Klebsiella pneumoniae	Klebsiella pneumoniae, Acinetobacter baumannii	Partly matched
330	F	73	/	ID	Intracranial infection	/	CSF	Neg	Neg	/
331	M	41	HT	ID	Pulmonary infection	Virus	BALF	Human herpesvirus 6A	Neg	/
332	M	53	HT	ID	Pulmonary infection	Virus	BALF	Leptosvirus	Neg	/
333	M	51	/	ID	Skin and soft tissue infection	Bacteria	Pus	Vibrio vulnificus	Neg	/
334	M	66	HT	ID	Blood stream infection	/	Blood	Neg	Neg	/
335	F	25	/	ID	Skin and soft tissue infection	Bacteria	Pus	Sphingomonas spinosa	Neg	/
336	M	49	/	ID	Pulmonary infection	Chlamydia	BALF	Chlamydia psittaci	Neg	/
337	F	62	/	NID	/	/	BALF	Neg	Neg	/
338	F	50	/	ID	Pulmonary infection	Bacteria	BALF	Burkholderia polyphagia	Neg	/
339	F	57	HT	ID	Pulmonary infection	Bacteria	BALF	Streptococcus pharyngitis	Neg	/
340	M	65	/	ID	Pulmonary infection	Bacteria	BALF	Klebsiella pneumoniae	Klebsiella pneumoniae	Matched
341	F	30	RD	ID	Skin and soft tissue infection	/	Pus	Neg	Neg	/
342	M	54	/	ID	Pulmonary infection	Fungi	BALF	Candida albicans	Neg	/
343	F	44	/	ID	Blood stream infection	Bacteria	Blood	Legionella pneumophila	Neg	/

No	Sex	Age (y)	Specific group	Infection status	Infection sites	Pathogen type	Specimens for mNGS	mNGS	TDM	Matching degree
344	M	62	/	ID	Pulmonary infection	Bacteria, Fungi	BALF	Pseudomonas aeruginosa, Klebsiella Michigan, Candida	Pseudomonas aeruginosa, Candida	Partly matched
345	M	70	RD	ID	Pulmonary infection	Virus	BALF	Neg	Influenza A virus	/
346	F	46	RD	ID	Pulmonary infection	/	BALF	Neg	Neg	/
347	M	60	/	ID	Pulmonary infection, Pleura infection	/	Pleural fluid	Neg	Neg	/
348	M	50	/	ID	Pulmonary infection, Blood stream infection	Bacteria, Fungi	BALF	Acinetobacter baumannii, Elizabethania, Stenotrophomonas maltophilia, Cryptococcus neoformans, Pneumocystis Yeyeri	Cryptococcus neoformans	Partly matched
349	M	53	/	ID	Pulmonary infection	/	Unclassified	Neg	Neg	/
350	M	23	HT	ID	Blood stream infection	/	Blood	Neg	Neg	/
351	M	80	/	ID	Pulmonary infection	Fungi	BALF	Aspergillus	Aspergillus	Matched
352	F	66	HT	ID	Pulmonary infection	Fungi	BALF	Aspergillus fumigatus	Neg	/
353	M	51	/	ID	IE	Bacteria	Tissue	Granulicatella adiacens, Streptococcus infantarius	Neg	/
354	M	64	/	NID	/	/	BALF	Neg	Neg	/
355	M	66	HT	ID	Blood stream infection	/	Blood	Neg	Neg	/
356	F	58	RD	ID	Pulmonary infection	Bacteria	BALF	Serratia marcescens	Chryseobacterium indologenes	Mismatched
357	M	65	/	NID	/	/	BALF	Neg	Neg	/
358	M	59	/	ID	Blood stream infection	Bacteria	Blood	Pseudomonas	Neg	/

No	Sex	Age (y)	Specific group	Infection status	Infection sites	Pathogen type	Specimens for mNGS	mNGS	TDM	Matching degree
359	F	88	/	ID	Pulmonary infection, Pleura infection	Bacteria	Pleural fluid	Intermediate streptococcus	Neg	/
360	F	40	/	ID	Blood stream infection	Bacteria	Blood	Intermediate streptococcus	Neg	/
361	M	80	/	ID	Pulmonary infection	Fungi	BALF	Aspergillus flavus, Aspergillus oryzae	Neg	/
362	F	61	RD	ID	Pulmonary infection	Bacteria	BALF	Staphylococcus aureus	Staphylococcus aureus	Matched
363	F	50	HT	ID	Blood stream infection	Virus	Blood	Human herpesvirus 3	Neg	/
364	M	57	/	ID	Blood stream infection	Bacteria	Blood	Klebsiella pneumoniae	Neg	/
365	M	71	HT	ID	Pulmonary infection	Fungi	BALF	Aspergillus fumigatus	Neg	/
366	F	71	/	ID	Pulmonary infection	/	BALF	Neg	Neg	/
367	M	23	RD	ID	Pulmonary infection	Bacteria, NTM	BALF	Mycobacterium abscessum, Acinetobacter baumannii, Elizabethania pacifica, Elizabethania anopheles	Acinetobacter baumannii	Partly matched
368	M	65	/	ID	Pulmonary infection	MTB	BALF	MTB	Neg	/
369	F	31	RD	ID	Pulmonary infection	/	BALF	Neg	Neg	/
370	M	66	RD	ID	Pulmonary infection	Bacteria, Fungi, NTM	BALF	Cyanobacteria marneffei, Candida albicans, Aspergillus fumigatus, Mycobacterium intracellular, Mycobacterium chimeratum	Candida albicans	Partly matched
371	F	56	/	ID	Pulmonary infection	NTM	BALF	Intracellular Mycobacterium, Mycobacterium Kansas	Neg	/

No	Sex	Age (y)	Specific group	Infection status	Infection sites	Pathogen type	Specimens for mNGS	mNGS	TDM	Matching degree
372	M	69	HT	ID	Intracranial infection	Bacteria, Fungi	CSF	Nocardia mallei, Aspergillus terreus	Neg	/
373	M	60	RD	ID	Pulmonary infection	/	BALF	Neg	Neg	/
374	M	81	RD	ID	Pulmonary infection	Bacteria	BALF	Neg	Escherichia coli	/
375	F	54	HT	ID	Blood stream infection	/	Blood	Neg	Neg	/
376	M	33	HT	ID	Blood stream infection	/	Blood	Neg	Neg	/
377	M	74	HT	ID	Blood stream infection	/	Blood	Neg	Neg	/
378	M	66	HT	ID	Blood stream infection	/	Blood	Neg	Neg	/
379	F	59	HT	ID	Blood stream infection	/	Blood	Neg	Neg	/
380	F	60	HT, RD	NID	/	/	CSF	Neg	Neg	/
381	M	65	RD	Unkown	/	/	BALF	Pos	Pos	/
382	F	61	HT	ID	Blood stream infection	/	Blood	Neg	Neg	/
383	M	56	/	ID	Pulmonary infection	Fungi	BALF	Aspergillus oryzae, Aspergillus flavus	Neg	/
384	M	88	/	NID	/	/	Pleural fluid	Neg	Neg	/
385	M	56	/	ID	Pulmonary infection	/	BALF	Neg	Neg	/
386	F	63	/	ID	Pulmonary infection	NTM	BALF	Intracellular mycobacterium	Neg	/
387	M	52	/	ID	Pulmonary infection	Fungi, NTM	BALF	Aspergillus fumigatus, Mycobacterium kansasii	Neg	/
388	M	54	HT	ID	Blood stream infection	Bacteria, Fungi	Blood	Pseudomonas aeruginosa, Rhizopus microsporus	Acinetobacter baumannii	Mismatched

No	Sex	Age (y)	Specific group	Infection status	Infection sites	Pathogen type	Specimens for mNGS	mNGS	TDM	Matching degree
389	F	77	/	ID	Pulmonary infection, Blood stream infection	Bacteria	BALF	Pseudomonas aeruginosa, Serratia marcescens, Elizabethania pacifica, Elizabethania anopheles, Burkholderia neoonion, Acinetobacter baumannii	Klebsiella pneumoniae	Mismatched
390	M	64	RD	ID	Pulmonary infection	Bacteria, Fungi	BALF	Stenotrophomonas maltophilia, Cryptococcus neoformans	Neg	/
391	F	54	HT	ID	Intracranial infection	Virus	CSF	Human herpesvirus 5	Neg	/
392	F	51	/	ID	Pulmonary infection	Fungi	BALF	Cryptococcus neoformans	Cryptococcus neoformans	Matched
393	F	66	HT, RD	ID	Pulmonary infection	Bacteria	BALF	Staphylococcus aureus	Staphylococcus aureus	Matched
394	M	63	/	ID	Pulmonary infection	Bacteria, MTB	Unclassified	Klebsiella pneumoniae, MTB	Klebsiella pneumoniae, Acid-fast bacilli	Matched
395	F	71	/	NID	/	/	BALF	Pos	Pos	/
396	M	72	/	NID	/	/	CSF	Neg	Neg	/
397	M	66	/	ID	Pulmonary infection	Bacteria	BALF	Acinetobacter baumannii	Acinetobacter baumannii	Matched
398	F	49	HT	ID	Pulmonary infection	Bacteria, Virus	BALF	Acinetobacter baumannii, Human herpesvirus type 1	Acinetobacter baumannii	Partly matched
399	M	66	/	Unkown	/	/	CSF	Neg	Pos	/
400	M	76	/	Unkown	/	/	CSF	Neg	Pos	/
401	M	67	/	ID	Pulmonary infection	Bacteria, Fungi	BALF	Fluorobacterium Goleman, Aspergillus flavus, Aspergillus oryzae	Neg	/
402	M	68	/	ID	Pulmonary infection	Fungi	BALF	Candida albicans	Candida albicans	Matched

No	Sex	Age (y)	Specific group	Infection status	Infection sites	Pathogen type	Specimens for mNGS	mNGS	TDM	Matching degree
403	M	58	HT	ID	Blood stream infection	Virus	Blood	Leptosvirus	Neg	/
404	F	62	/	ID	Pulmonary infection	Fungi, NTM	BALF	Aspergillus fumigatus, Mycobacterium avium	Neg	/
405	F	77	RD	ID	Abdominal infection	/	Ascitic fluid	Neg	Neg	/
406	M	40	/	ID	Blood stream infection	/	Blood	Neg	Neg	/
407	M	69	/	NID	/	/	BALF	Neg	Neg	/
408	M	51	/	ID	Pulmonary infection	Fungi	BALF	Cryptococcus neoformans	Neg	/
409	M	72	HT	ID	Pulmonary infection	/	Tissue	Neg	Neg	/
410	F	83	/	ID	Blood stream infection	/	Blood	Neg	Neg	/
411	M	84	/	ID	Pulmonary infection	Bacteria, Fungi	BALF	Stenotrophomonas maltophilia, Aspergillus fumigatus	Stenotrophomonas maltophilia	Partly matched
412	F	60	HT	ID	Pulmonary infection	Fungi, NTM	BALF	Mycobacterium abscessu	Aspergillus	Mismatched
413	F	38	/	ID	Pulmonary infection	Bacteria	BALF	Legionella martensii, Legionella pneumophila	Neg	/
414	M	56	RD	ID	Mumpus	Bacteria	Pus	Lesheplevobacteria, Prevotella oral, Intermediate streptococcus	Neg	/
415	F	70	/	ID	Pulmonary infection	/	BALF	Neg	Neg	/
416	F	60	/	ID	Pulmonary infection	NTM	BALF	mycobacteria intracellular	Neg	/
417	F	65	/	ID	Pulmonary infection	NTM	BALF	mycobacteria intracellular	Neg	/
418	M	52	/	ID	Pulmonary infection	Bacteria	BALF	Serratia marcescens, Elizabethania pacifica	Neg	/

No	Sex	Age (y)	Specific group	Infection status	Infection sites	Pathogen type	Specimens for mNGS	mNGS	TDM	Matching degree
419	M	61	/	ID	Pulmonary infection, Pleura infection	/	Pleural fluid	Neg	Neg	/
420	M	45	/	ID	Pulmonary infection	Bacteria, Fungi	BALF	Acinetobacter baumannii, Elizabethania pacifica, Elizabethania anopheles, Aspergillus fischeri	Neg	/
421	M	26	/	ID	Pulmonary infection	NTM	BALF	Mycobacterium abscessum	Neg	/
422	F	65	/	ID	Pulmonary infection	Fungi	BALF	Aspergillus	Aspergillus	Matched
423	M	46	/	ID	Pulmonary infection	/	BALF	Neg	Neg	/
424	M	34	/	Unkown	/	/	BALF	Neg	Neg	/
425	F	61	/	ID	Pulmonary infection	Fungi	BALF	Neg	Aspergillus	/
426	F	53	/	ID	Blood stream infection	/	Blood	Neg	Neg	/
427	M	39	HT, RD	ID	Blood stream infection	Bacteria	Blood	Neg	Xanthomonas	/
428	F	82	/	ID	Intracranial infection	Bacteria	CSF	Nocardia mallei	Neg	/
429	F	30	/	ID	Intracranial infection	/	CSF	Neg	Neg	/
430	M	62	/	ID	Pulmonary infection	/	BALF	Neg	Neg	/
431	F	63	/	ID	Pulmonary infection	/	BALF	Neg	Neg	/
432	F	25	/	NID	/	/	Pericardial fluid	Neg	Neg	/
433	M	79	/	ID	Pulmonary infection	Fungi	BALF	Aspergillus fumigatus	Neg	/
434	M	58	HT	ID	Pulmonary infection	/	BALF	Neg	Neg	/
435	F	59	/	ID	Pulmonary infection	/	BALF	Neg	Neg	/

Abbreviations: mNGS, metagenomic next-generation sequencing; TDM, traditional diagnostic method; M, male; F, female; RD, Rheumatic disease; HT, hematological tumor; IE, Infective endocarditis; NTM, Nontuberculous mycobacteria; MTB, Mycobacterium tuberculosis; BALF, bronchial alveolar lavage fluid; CSF, cerebrospinal fluid; Neg, negative; Pos, positive

Supplementary Table 2 Analysis of patients with co-infection

No	Sex	Age (y)	Specific group	Infection status	Infection sites	Pathogen type	Specimens for mNGS	mNGS	TDM	Matching degree
7	F	67	RD	ID	Pulmonary infection, Intracranial infection	Fungi, NTM	BALF, CSF	Aspergillus fumigatus, Mycobacterium abscessum	Neg	/
11	F	69	/	ID	Pulmonary infection	Bacteria, Virus	BALF	Haemophilus influenzae, Streptococcus pneumoniae, Human herpesvirus 1	Neg	/
14	F	49	/	ID	Pulmonary infection	Bacteria, Mycoplasma, NTM	BALF	Haemophilus influenzae, Staphylococcus aureus, Mycoplasma hominis, Ureaplasma parvum, Mycobacterium abscessum	Neg	/
51	F	63	HT	ID	Pulmonary infection	Bacteria, Virus	BALF	Pseudomonas aeruginosa, Human herpesvirus 5	Neg	/
55	M	50	/	ID	Pulmonary infection	Fungi, MTB	BALF	Aspergillus fumigatus	Aspergillus, Acid-fast bacilli	Partly matched
60	F	32	/	ID	Pulmonary infection	Fungi, MTB	Tissue	MTB	Aspergillus, MTB	Partly matched
101	F	39	/	ID	Blood stream infection	Bacteria, Virus	Blood	Stenotrophomonas maltophilia, Human herpesvirus 1	Neg	/
117	M	62	/	ID	Pulmonary infection	Bacteria, Fungi, NTM	BALF	Klebsiella pneumoniae, Mycobacteria intracellular, Mycobacterium chimerae	Aspergillus fumigatus	Partly matched
148	F	78	/	ID	Pulmonary infection	Fungi, MTB	BALF	Cryptococcus neoformans, MTB	Acid-fast bacilli	Partly matched

No	Sex	Age (y)	Specific group	Infection status	Infection sites	Pathogen type	Specimens for mNGS	mNGS	TDM	Matching degree
187	M	65	/	ID	Pulmonary infection	Fungi, parasite	BALF	Pneumocystis yersinii	Cryptococcus lorentus	Mismatched
190	F	54	/	ID	Pulmonary infection	Fungi, NTM	BALF	Aspergillus, Mycobacterium avium	Aspergillus	Partly matched
203	M	57	/	ID	Pulmonary infection	Bacteria, Fungi	BALF	Stenotrophomonas maltophilia, Candida albicans	Neg	/
210	F	25	HT	ID	Blood stream infection	Virus, Fungi	Blood	Human herpesvirus 4	Aspergillus	Mismatched
217	M	64	/	ID	Pulmonary infection	Bacteria, Fungi	BALF	Haemophilus influenzae, Klebsiella pneumoniae, Aspergillus	Klebsiella pneumoniae	Partly matched
240	M	91	/	ID	Pulmonary infection	Bacteria, NTM	BALF	Acinetobacter baumannii, Pseudomonas aeruginosa, Mycobacterium abscessum	Acinetobacter baumannii	Partly matched
253	M	74	/	ID	Pulmonary infection	Virus, Fungi	BALF	Human herpesvirus 4, Aspergillus flavus	Neg	/
263	M	33	/	ID	Pulmonary infection	Bacteria, Virus, NTM, Parasite	BALF	Serratia marcescens, Mycobacterium abscessum, Pneumocystis yersinii, Human herpesvirus type 4	Neg	/
277	F	63	/	ID	Pulmonary infection	Bacteria, NTM	BALF	Stenotrophomonas maltophilia, Mycobacterium avium	Neg	/

No	Sex	Age (y)	Specific group	Infection status	Infection sites	Pathogen type	Specimens for mNGS	mNGS	TDM	Matching degree
300	F	75	/	ID	Pulmonary infection, Blood stream infection	Bacteria, NTM	BALF, Blood	Burkholderia neoonion, Elizabethania, Mycobacterium abscessum	Elizabethania	Partly matched
313	F	89	/	ID	Pulmonary infection	Bacteria, Fungi	BALF	Stenotrophomonas maltophilia, Pseudomonas aeruginosa, Aspergillus fumigatus	Pseudomonas aeruginosa	Partly matched
318	M	73	/	ID	Pulmonary infection	Bacteria, Fungi	BALF	Acinetobacter baumannii, Aspergillus fumigatus, Aspergillus oryzae, Aspergillus flavus	Acinetobacter baumannii	Partly matched
319	F	66	/	ID	Pulmonary infection	Fungi, NTM	BALF	Mycobacterium kansasii, Aspergillus terreus, Aspergillus fumigatus	Acid-fast bacilli, Fungus	Matched
344	M	62	/	ID	Pulmonary infection	Bacteria, Fungi	BALF	Pseudomonas aeruginosa, Klebsiella Michigan, Candida	Pseudomonas aeruginosa, Candida	Partly matched
348	M	50	/	ID	Pulmonary infection, Blood stream infection	Bacteria, Fungi	BALF	Acinetobacter baumannii, Elizabethania, Stenotrophomonas maltophilia, Cryptococcus neoformans, Pneumocystis Yeyeri	Cryptococcus neoformans	Partly matched

No	Sex	Age (y)	Specific group	Infection status	Infection sites	Pathogen type	Specimens for mNGS	mNGS	TDM	Matching degree
367	M	23	RD	ID	Pulmonary infection	Bacteria, NTM	BALF	Mycobacterium abscessum, Acinetobacter baumannii, Elizabethania pacifica, Elizabethania anopheles	Acinetobacter baumannii	Partly matched
370	M	66	RD	ID	Pulmonary infection	Bacteria, Fungi, NTM	BALF	Cyanobacteria marneffei, Candida albicans, Aspergillus fumigatus, Mycobacterium intracellular, Mycobacterium chimeratum	Candida albicans	Partly matched
372	M	69	HT	ID	Intracranial infection	Bacteria, Fungi	CSF	Nocardia mallei, Aspergillus terreus	Neg	/
387	M	52	/	ID	Pulmonary infection	Fungi, NTM	BALF	Aspergillus fumigatus, Mycobacterium kansasii	Neg	/
388	M	54	HT	ID	Blood stream infection	Bacteria, Fungi	Blood	Pseudomonas aeruginosa, Rhizopus microsporus	Acinetobacter baumannii	Mismatched
390	M	64	RD	ID	Pulmonary infection	Bacteria, Fungi	BALF	Stenotrophomonas maltophilia, Cryptococcus neoformans	Neg	/
394	M	63	/	ID	Pulmonary infection	Bacteria, MTB	Unclassified	Klebsiella pneumoniae, MTB	Klebsiella pneumoniae, Acid-fast bacilli	Matched
398	F	49	HT	ID	Pulmonary infection	Bacteria, Virus	BALF	Acinetobacter baumannii, Human herpesvirus type 1	Acinetobacter baumannii	Partly matched
401	M	67	/	ID	Pulmonary infection	Bacteria, Fungi	BALF	Fluorobacterium Goleman, Aspergillus flavus, Aspergillus oryzae	Neg	/

No	Sex	Age (y)	Specific group	Infection status	Infection sites	Pathogen type	Specimens for mNGS	mNGS	TDM	Matching degree
404	F	62	/	ID	Pulmonary infection	Fungi, NTM	BALF	Aspergillus fumigatus, Mycobacterium avium	Neg	/
411	M	84	/	ID	Pulmonary infection	Bacteria, Fungi	BALF	Stenotrophomonas maltophilia, Aspergillus fumigatus	Stenotrophomonas maltophilia	Partly matched
412	F	60	HT	ID	Pulmonary infection	Fungi, NTM	BALF	Mycobacterium abscessu	Aspergillus	Mismatched
420	M	45	/	ID	Pulmonary infection	Bacteria, Fungi	BALF	Acinetobacter baumannii, Elizabethania pacifica, Elizabethania anopheles, Aspergillus fischeri	Neg	/

Abbreviations: mNGS, metagenomic next-generation sequencing; TDM, traditional diagnostic method; M, male; F, female; RD, Rheumatic disease; HT, hematological tumor; IE, Infective endocarditis; NTM, Nontuberculous mycobacteria; MTB, Mycobacterium tuberculosis; BALF, bronchial alveolar lavage fluid; CSF, cerebrospinal fluid; Neg, negative; Pos, positive.

Supplementary Table 3 Analysis of patients with intracranial infection

No	Sex	Age (y)	Specific group	Infection status	Infection sites	Pathogen type	Specimens for mNGS	mNGS	TDM	Matching degree
7	F	67	RD	ID	Pulmonary infection, Intracranial infection	Fungi, NTM	BALF, CSF	Aspergillus fumigatus, Mycobacterium abscessum	Neg	/
66	F	44	/	ID	Intracranial infection	Bacteria	CSF	Streptococcus constellatus	Neg	/
67	F	19	/	ID	Intracranial infection	Virus	CSF	Human herpesvirus 1	Neg	/
68	M	82	/	ID	Intracranial infection	Virus	CSF	Human herpesvirus 3	Neg	/
69	M	73	/	ID	Intracranial infection	Virus	CSF	Human herpesvirus 3	Human herpesvirus 3	Matched
70	M	20	/	ID	Intracranial infection	Virus	CSF	Human herpesvirus 3	Human herpesvirus 3	Matched
71	M	38	HT	ID	Intracranial infection	/	CSF	Neg	Neg	/
72	M	63	/	ID	Intracranial infection	/	CSF	Neg	Neg	/
73	F	76	/	ID	Intracranial infection	/	CSF	Neg	Neg	/
74	M	55	/	ID	Intracranial infection	Bacteria	CSF	Neg	Acinetobacter baumannii, Stenotrophomonas maltophilia	/
76	M	71	/	ID	Intracranial infection	/	CSF	Neg	Neg	/
77	F	47	/	ID	Intracranial infection	/	CSF	Neg	Neg	/
78	F	26	/	ID	Intracranial infection	/	CSF	Neg	Neg	/
79	M	30	/	ID	Intracranial infection	/	CSF	Neg	Neg	/
80	F	58	/	ID	Intracranial infection	/	CSF	Neg	Neg	/
81	M	29	/	ID	Intracranial infection	/	CSF	Neg	Neg	/
82	M	50	/	ID	Intracranial infection	/	CSF	Neg	Neg	/
116	M	68	/	ID	Intracranial infection	MTB	CSF	Neg	MTB	/
133	M	35	/	ID	Intracranial infection	Bacteria	CSF	Acinetobacter baumannii	Neg	/
140	F	53	/	ID	Intracranial infection	Virus	CSF	Human herpesvirus 4	Human herpesvirus 4	Matched
147	F	59	/	ID	Intracranial infection	Bacteria	CSF	Streptococcus gordonii	Neg	/

No	Sex	Age (y)	Specific group	Infection status	Infection sites	Pathogen type	Specimens for mNGS	mNGS	TDM	Matching degree
156	M	46	/	ID	Intracranial infection	Virus	CSF	Human herpesvirus 4	Neg	/
175	M	53	/	ID	Intracranial infection	Bacteria	CSF	Lactococcus canadensis, Streptococcus gordon	Acinetobacter baumannii	Mismatched
177	F	52	/	ID	Intracranial infection	Bacteria	CSF	Neg	Citrobacter braakii	/
184	F	53	/	ID	Intracranial infection	/	CSF	Neg	Neg	/
191	M	25	/	ID	Intracranial infection	Fungi	CSF	Cryptococcus neoformans	Cryptococcus neoformans	Matched
201	M	59	/	ID	Intracranial infection	/	CSF	Neg	Neg	/
202	F	82	/	ID	Intracranial infection	MTB	CSF	MTB	Neg	/
205	M	51	/	ID	Intracranial infection	/	CSF	Neg	Neg	/
211	M	70	/	ID	Pulmonary infection, Intracranial infection	/	BALF, CSF	Neg	Neg	/
213	F	29	/	ID	Intracranial infection	/	CSF	Neg	Neg	/
215	M	44	/	ID	Intracranial infection	/	CSF	Neg	Neg	/
216	M	22	/	ID	Intracranial infection	/	CSF	Neg	Neg	/
223	M	37	/	ID	Intracranial infection	/	CSF	Neg	Neg	/
233	F	34	/	ID	Intracranial infection	Bacteria	CSF	Acinetobacter baumannii	Neg	/
235	M	35	/	ID	Intracranial infection	Virus	CSF	Human herpesvirus 3	Neg	/
242	M	65	/	ID	Intracranial infection	/	CSF	Neg	Neg	/
254	F	66	/	ID	Intracranial infection	/	CSF	Neg	Neg	/
258	M	41	/	ID	Intracranial infection	/	CSF	Neg	Neg	/
280	M	55	/	ID	Intracranial infection	/	CSF	Neg	Neg	/
322	M	51	/	ID	Intracranial infection	/	CSF	Neg	Neg	/
323	F	39	RD	ID	Intracranial infection	/	CSF	Neg	Neg	/
324	M	36	/	ID	Intracranial infection	/	CSF	Neg	Neg	/

No	Sex	Age (y)	Specific group	Infection status	Infection sites	Pathogen type	Specimens for mNGS	mNGS	TDM	Matching degree
330	F	73	/	ID	Intracranial infection	/	CSF	Neg	Neg	/
372	M	69	HT	ID	Intracranial infection	Bacteria, Fungi	CSF	Nocardia mallei, Aspergillus terreus	Neg	/
391	F	54	HT	ID	Intracranial infection	Virus	CSF	Human herpesvirus 5	Neg	/
428	F	82	/	ID	Intracranial infection	Bacteria	CSF	Nocardia mallei	Neg	/
429	F	30	/	ID	Intracranial infection	/	CSF	Neg	Neg	/

Abbreviations: mNGS, metagenomic next-generation sequencing; TDM, traditional diagnostic method; M, male; F, female; RD, Rheumatic disease; HT, hematological tumor; IE, Infective endocarditis; NTM, Nontuberculous mycobacteria; MTB, Mycobacterium tuberculosis; BALF, bronchial alveolar lavage fluid; CSF, cerebrospinal fluid; Neg, negative; Pos, positive
